# Physical Activity Levels in Women Attending Breast Screening, Receiving Chemotherapy and Post-Breast Cancer Treatment; A Cross-Sectional Study

**DOI:** 10.3390/ijerph110505487

**Published:** 2014-05-20

**Authors:** Ian M. Lahart, George S. Metsios, Alan M. Nevill, Amtul R. Carmichael

**Affiliations:** 1 Faculty of Education, Health and Wellbeing, University of Wolverhampton, Walsall Campus, Gorway Road, Walsall, WS1 3BD, UK; E-Mails: I.Lahart@wlv.ac.uk (I.L.); G.Metsios@wlv.ac.uk (G.M.); a.m.nevill@wlv.ac.uk (A.N.); 2 Department of General Surgery, Russells Hall Hospital, Dudley DY12HQ, UK

**Keywords:** breast cancer, physical activity, chemotherapy

## Abstract

*Background*: A lack of physical activity (PA) is a well-recognised risk factor in the development of breast cancer (BC) and evidence-base research on the impact of PA on BC survival is consolidating. However, evidence reveals that BC survivors have low levels of PA, suggesting the need of targeted interventions to enhance the PA behaviour of BC survivors. Unfortunately, there is lack of data from the UK about the PA behaviours of women at various stages of diagnosis and treatment of BC. Therefore, the aim of the present study was to assess PA levels in women at different stages of BC pathway. *Patients and Methods*: A convenient sample of patients was selected at various stages of presentation and treatment of BC. Patients attending for breast screening for NHSBSP (n = 188), post-operative patients attending for chemotherapy (n = 41) and BC patients within one year’s post-treatment (n = 80) were invited to take part in this cross-sectional study. *Results*: Based on the odds ratio, the likelihood of a chemotherapy participant not meeting PA guidelines (*i.e.*, being in the low activity category) were three times higher than the odds of a NHSBPS attendee not meeting PA guidelines, and compared to post-treatment participants, the chemotherapy patient’s odds of not meeting PA guidelines was four times higher. The odds of NHSBPS attendees being in the high activity category compared to the moderate category were three times higher than that of a post-treatment participant. *Conclusions*: The current study suggests the need to establish robust PA interventions to enhance the PA behaviour of breast cancer survivors.

## 1. Introduction

Worldwide, breast cancer (BC) is the most frequently diagnosed cancer and the leading cause of cancer death among females [1]. In the UK, female BC had the highest incidence rate of all cancers, with an average European AS rate of 124.2 cases per 100,000 population each year between 2007 and 2009 [2]. As a large consequence of early detection and improved treatment strategies, UK BC mortality rates are falling and in turn survival rates are improving [3] and thus there are now more BC survivors than ever before. However, due to the chronic side-effects of BC treatment, survivors may require diagnostic, therapeutic, supportive or palliative services many years post-diagnosis, which poses a major burden to already stretched healthcare resources. Therefore, interventions are required that can not only reduce the risk of BC recurrence, but in addition can benefit BC survivors.

A lack of physical activity (PA) is a well-recognised risk factor in the development of BC and evidence on the impact of PA on BC survival is consolidating [4]. In prospective studies, an average 25% reduction in the risk of developing BC was seen in the most physically active women compared to the least active women while this risk reduction is shown to be dose-dependent [5].

Evidence also suggests that achieving recommended levels of PA can improve disease specific mortality after the diagnosis of BC [6]. In addition, a recent Cochrane review demonstrated that PA can significantly improve the quality of life of women treated for BC [7]. However, despite these positive effects of PA, evidence reveals that BC survivors have low levels of PA and many women decrease their PA following diagnosis [8,9]. As such, targeting behaviours that may increase levels of PA may be beneficial for BC patients [10]. Unfortunately, there is lack of data from the UK about the PA behaviours of women at various stages of diagnosis and treatment of BC. Therefore, the aim of the present study was to assess PA levels in women at different stages of breast cancer pathway.

## 2. Patients and Methods

A convenience sample of patients was selected at various stages of presentation and treatment of BC. Patients attending for breast screening for the National Health Service Breast Screening programme (NHSBSP) (n = 188), post-operative patients attending for chemotherapy (n = 41) and BC patients after one year’s post-treatment (n = 80) were invited to take part in this cross-sectional study. Participants within each group completed an assessment which included demographic and PA questionnaires and anthropometric measurements. The height and weight of patients receiving chemotherapy and the post-treatment patients were measured directly in breast clinics at Russells Hall Hospital, Dudley. The assessment of the BC screening attendees was carried out in a mobile screening unit located in Sandwell, West Midlands, and the self-reported height and weight were taken from these participants. Participant’s height and weight were then used to calculate BMI. Each participant completed the validated long form International PA Questionnaire (IPAQ) [11]. The IPAQ assessed moderate and vigorous PA in four life domains: occupational, active transportation, domestic and leisure-time, and walking in the occupational, active transport and leisure-time domains. IPAQ was administered during face-to-face interviews by specifically trained researchers [12].

PA categories were classified using the recommendations outlined in the IPAQ manual [13]. Using MET-min·wk^−1^ as a means to combine aerobic activities of different types and intensities into a single measure of amount of activity, the IPAQ PA categories allow the categorisation of individuals into those who are meeting the current recommended PA guidelines (*i.e.*, a minimum of 150 min of moderate-intensity physical activity per week) and those who are not (*i.e.*, <150 min·wk) [14]. The IPAQ categorises PA into low (not achieving recommended levels), moderate and high activity categories. Based on recent recommendations about IPAQ [15], we included separate analysis using either moderate-to-high or high activity category alone to identify those meeting recommended PA guidelines.

## 3. Statistical Analysis

The normal distribution of the studied variables was assessed via the Kolmogorov-Smirnov tests of normality; data that were normally distributed were reported as mean (±*s*), while data not normally distributed were expressed as median (interquartile range).

Height differences (the only normally distributed variable) between groups were analysed with one-way Analysis of Variance (ANOVA) using Bonferoni *post-hoc* analysis for individual comparisons. Kruskal-Wallis tests were performed on all of the other variables of interest because they were not normally distributed. These tests were analysed *post hoc* using the critical difference method as described by Siegel and Castellan [16]. Physical activity categorical data were expressed as number of participants and percentages and were analysed via chi-square analysis. The level of significance was set at *p* < 0.05 for between groups analysis.

We first compared the number of participants in the three PA categories between the three groups. If significant associations were found, we planned a number of comparisons to identify which comparisons were significant. There were nine planned comparisons, including the number of participants in the low activity category *versus* moderate activity plus high activity category (*i.e.*, those meeting recommended physical activity guidelines), number of participants in the high activity category *versus* low activity plus moderate activity category, and the number of participants in the moderate activity *versus* high activity category in the NHSBSP participants *versus* the chemotherapy patients, the NHSBSP participants *versus* the post-treatment patients, and the chemotherapy patients *versus* the post-treatment patients. In order to counter the inflation of type I error caused by multiple comparisons, a Bonferoni correction was applied to the alpha level. In this case we planned nine comparisons, which gave us an alpha level of *p* < 0.0056 (*i.e.*, 0.05/9 = 0.0056). Statistical analyses were performed using IBM^®^ Statistical Package for Social Sciences^®^ for Windows version 20.0 (IBM, Armonk, NY, USA).

## 4. Results

Characteristics of the participants in each group are presented in Table 1. In brief, there was a slightly larger percentage (42%) of participants with a normal BMI (18–24.9 kg·m^2^) in the NHSBPS attendees compared to the chemotherapy and post-treatment group. All of the groups had a similar majority of white British participants. Unsurprisingly, given the age at which women are invited to breast screening, there were less premenopausal women in the NHSBPS group compared to the other two groups. In addition, a larger percentage of the post-treatment group were current or previous smokers (although the number of current smokers in this group was small, 5 out of 80, or 6%), compared to the other two groups.

**Table 1:** Anthropometric, demographic and lifestyle characteristics of the participants.

Variable	NHSBPS (n = 188)		Chemotherapy (n = 41)		Post-treatment (n = 80)	
	n	%	n	%	n	%
BMI (kg·m^2^) *						
• Obese (BMI ≥ 30)	36	25	11	26	22	28
• Overweight (BMI = 25–29.9)	46	32	9	22	29	36
• Normal (BMI = 18–24.9)	60	42	13	31	28	35
• Underweight (BMI ≤ 18)	0	0	0	0	1	1
• Missing	46	24	8	20	0	0
Ethnic Origin						
• White British	178	95	40	98	76	95
• Pakistani	1	0	1	2	0	0
• Indian	3	2	0	0	0	0
• Black Caribbean	2	1	0	0	2	3
• White Irish	2	1	0	0	1	1
• Other white background	1	1	0	0	1	1
• Chinese	1	1	0	0	0	0
• Missing	0	0	0	0	0	0
Menopausal status **^†^**						
• Premenopausal	13	7	10	24	16	20
• Perimenopausal	15	8	7	17	NR	NR
• Postmenopausal	146	78	24	59	64	80
• Unsure	8	4	0	0	0	0
• Missing	6	3	0	0	0	0
Employment status						
• Full-time	130 **^ǂ^**	69	10	24	29	36
• Part-time	NR	NR	9	22	13	17
• Retired	48	25	8	20	21	26
• Unemployed	2	1	2	5	3	4
• Homemaker	1	1	4	10	8	10
• Other	0	0	0	0	3	4
• Missing	7	37	8	20	3	4
Smoking						
• Currently/Previously	23	12	6	15	33	42
• No	165	88	35	85	47	48

Notes: n = number, NR = not reported; * For NHSBSP participants BMI was computed from self-reported height and mass; **^†^** For post-treatment participants premenopausal status was determined as those participants who were currently or recently menstruating; **^ǂ^** Figure represents those participants who were employed, both part-time and full-time, no separate figures were available for each.

The descriptive statistics of the anthropometric and PA variables for each group are presented in Table 2. There were no significant differences in height (*p* = 0.077), body mass (*p* = 0.626) or BMI (*p* = 0.854) between the participant groups.

**Table 2:** Mean ± *s* or median (interquartile range) for anthropometric measures and physical activity (PA) variables for NHSBPS participants, breast cancer patients undergoing chemotherapy, and breast cancer patients within one-year post-treatment (PA in MET-min·wk^−1^ unless stated otherwise).

Variable	NHSBSP		Chemotherapy		Post-treatment			
	n	Median (IQR)	n	Median (IQR)	n	Median (IQR)	F/H Statistic	*p*-value
Height (m) mean ± *s*	179	1.63 ± 0.06	34	1.63 ± 0.07	80	1.61 (0.07)	3.494	0.077
Body mass (kg)	145	68.9 (19.6)	34	70.0 (21.6)	80	67.7 (14.2)	0.937	0.626
BMI (kg∙m^2^)	143	26.4 (7.3)	34	27.5 (5.3)	80	26.0 (6.1)	0.315	0.854
Overall PA	188	1689 (2345) *	41	933 (2127) **^†^**	80	1463 (1501) **^ǂ^**	12.286	0.002
Work PA	188	0 (0)	41	0 (0)	80	0 (0)	0.329	0.848
Active transport PA	188	66 (387)	41	50 (479)	80	132 (264)	1.133	0.567
Domestic PA	188	774 (1281) *	41	297 (897) **^†^**	80	541 (987) **^ǂ^**	14.52	0.001
Leisure PA	188	330 (1031) *	41	0 (314) **^†^**	80	350 (693)	11.253	0.004
Walk	188	396 (974)	41	297 (693)	80	396 (565)	0.838	0.658
Moderate PA	188	965 (1612) *	41	396 (1370) **^†^**	80	693 (1,060)	17.642	0.000
Vigorous PA	188	0 (0)	41	0 (0) **^†^**	80	0 (0)	6.059	0.048
Overall PA time (min)	188	420 (571) *	41	270 (602) **^†^**	80	416 (390)	7.366	0.025
Walk time (min)	188	120 (295)	41	90 (210)	80	120 (171)	0.838	0.658
Moderate PA time (min)	188	235 (300) *	41	120 (385) **^†^**	80	178 (297) **^ǂ^**	11.679	0.003
Vigorous PA time(min)	188	0 (0)	41	0 (0) **^†^**	80	0 (0)	5.992	0.049

Notes: n = number * Significantly higher in the NHSBPS group *vs.* the chemotherapy group; **^†^** Significantly lower in the chemotherapy group *vs.* the post-treatment group; **^ǂ^** Significantly lower than in the post-treatment group *vs.* the NHSBSP group.

**Figure 1:**
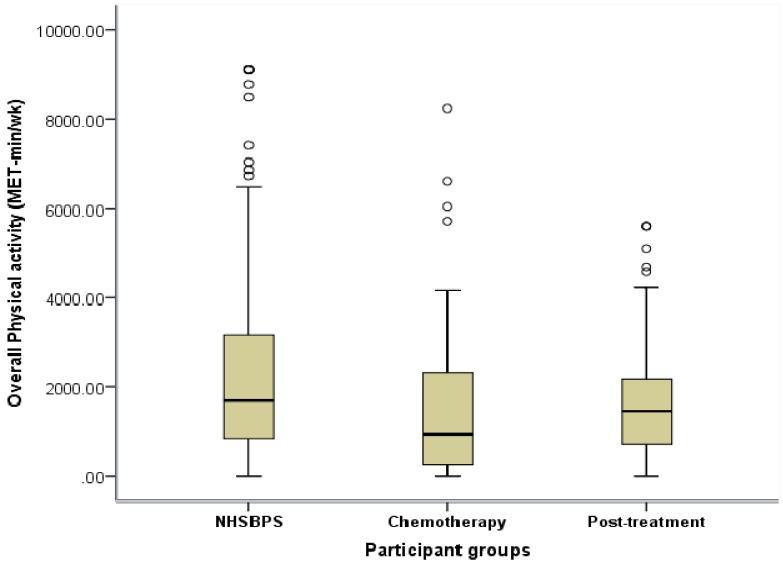
Box-plot for overall physical activity (MET-min·wk^−1^) of NHSBPS attendees, chemotherapy breast cancer patients and post-treatment breast cancer survivors.

Kruskal-Wallis tests revealed significant differences in overall (*p* < 0.01) (Figure 1), domestic (*p* < 0.01), leisure (*p* < 0.01), moderate (*p* < 0.01) and vigorous (*p* < 0.05) PA (MET-min·wk^−1^), and overall (*p* < 0.05), moderate (*p* < 0.01) and vigorous (*p* < 0.05) PA time (min) between the three groups.

*Post-hoc* analysis revealed significantly lower overall (*p* < 0.001), domestic (*p* < 0.001), leisure (*p* < 0.001) and moderate (*p* < 0.001) PA (MET- min·wk^−1^) and overall (*p* < 0.01) and moderate (*p* < 0.001) PA time (min) in the BC patients undergoing chemotherapy compared to the NHSBSP participant group. The chemotherapy participants also performed significantly less overall (*p* < 0.001), domestic (*p* < 0.001), leisure (*p* < 0.001), moderate (*p* < 0.05) and vigorous (*p* < 0.01) PA (MET-min·wk^−1^) and overall (*p* < 0.01), moderate (*p* < 0.01) and vigorous (*p* < 0.05) PA time (min) than the post-treatment participant group. The post-treatment BC survivors performed significantly less overall, domestic (*p* < 0.05) PA (MET- min·wk^−1^) and moderate (*p* < 0.05) PA time (min) than the NHSBSP participants.

**Figure 2:**
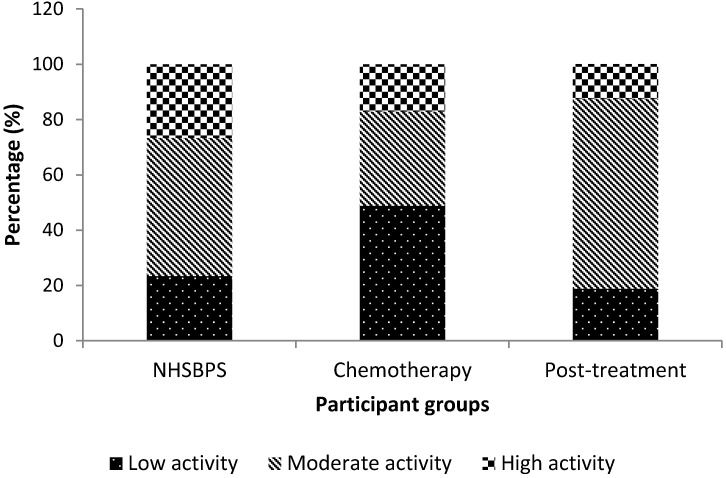
Percentage of participants in each physical activity category in the NHSBPS attendees, chemotherapy breast cancer patients and post-treatment breast cancer survivors.

According to IPAQ PA categories, there was a higher proportion of participants (20 out of 41) categorised as low activity in the chemotherapy participant group compared to the NHSBSP group (44/188) and the post-treatment group (15/80) (Figure 2). A greater proportion of participants in the NHSBPS participant group were in the high activity category (50/188) compared to the chemotherapy and post-treatment groups (7/41 and 10/80, respectively), while a larger proportion of the post-treatment group were categorised as moderate activity (55/80) compared to the NHSBSP and chemotherapy groups (14/41 and 94/188, respectively).

There was a significant association between the population groups and the categories of activity χ^2^ (4) = 22.887, *p* < 0.001. *Post-hoc* analysis with a Bonferoni correction applied (*p* < 0.0056) revealed that chemotherapy patients were more likely to be categorised as low activity (*i.e.*, not meeting recommended PA guidelines) than moderate or high category compared to the NHSBSP attendees (*p* = 0.001) and post-treatment BC patients (*p* = 0.001), while post-treatment patients were less likely to be categorised in the high category than the moderate category compared to the NHSBPS participants (*p* = 0.004). When we categorised those only in the high category as meeting recommended PA guidelines we found no association with participant groups.

Based on the odds ratio, the odds of a chemotherapy participant not meeting PA guidelines (*i.e.*, being in the low activity category) were 3.1 times higher than the odds of a NHSBPS attendee not meeting PA guidelines, and compared to post-treatment participants, the chemotherapy patient’s odds of not meeting PA guidelines was 4.1 times higher. The odds of NHSBPS attendees being in the high activity category compared to the moderate category were 2.9 times higher than that of a post-treatment participant.

## 5. Discussion and Conclusions

Results revealed that women receiving chemotherapy for BC performed significantly lower PA in a number of IPAQ domains compared to the women attending NHS breast screening, and BC patients who were within one year post-treatment. The post-treatment BC patients also performed significantly less PA compared to the breast screening participants. Chemotherapy patients were less likely to be meeting the recommended guidelines when compared to the other two groups. In addition, the post-treatment patients were less likely to be categorised as high activity compared to the HSBPS participants.

The finding of relatively low levels of PA in the two BC patient groups is supported by past research. Previous studies have reported low levels of PA in patients receiving chemotherapy [17,18], and have ascribed this finding largely to the side-effects of treatment, and treatment-related fatigue in particular [17]. Irwin and colleagues [9] surveyed the self-reported PA levels of 806 BC patients within three years post-diagnosis, and found that when household and gardening activities were excluded, only 32% of BC survivors achieved recommended levels of physical activity. Similarly, in the current study there were relatively few participants in the post-treatment group who reported high moderate-to-vigorous activity (*i.e.*, categorised as high activity).

Although, it was not possible to ascertain in this current study, previous studies comparing BC patients pre-diagnosis PA levels to their post-diagnosis levels, have found decreases in PA from pre- to post-treatment [8]. In a prospective cohort study [8] that compared the self-reported PA of 812 BC patients, reported an 11% decrease in total PA (h∙wk^-1^) from pre- to post-diagnosis in women treated for radiation and chemotherapy. Subsequent studies have also found a similar decrease in PA in the 12 months after diagnosis relative to before diagnosis [19]. These observed decreases have been attributed to persistent negative side effects of BC treatment including fatigue, nausea, and pain [20,21]. Consistent with the findings of the current study, the results of the above studies suggest that pre-BC diagnosis PA levels are higher than the PA levels of patients post-diagnosis.

However, while the general finding in the literature supports a decrease in PA from pre-diagnosis to during treatment to post-treatment, a recent finding from a Swedish study suggests that this may not be true for all women [22]. The authors observed that BC patients who had been PA before their cancer diagnosis and women who had received information about PA were more physically active during chemotherapy. Therefore, further research is required to establish how pre-breast cancer diagnosis PA along with other possible factors can mediate the levels of physical activity post-treatment.

Our study has several limitations. As is the case with all cross-sectional studies, it was not possible to attribute a temporal relationship between the group treatment status (*i.e*., breast screening, chemotherapy and post-treatment) and PA. That is, although we have found an association between PA levels and the treatment status of the participants, there is no evidence that the treatment status of participants was the cause of the reported PA. Furthermore, the cross-sectional design of the current study provided a “snap shot” of PA behaviour within the three study groups. Hence, it is possible that this “snap shot” of PA reported by the participants in each group were not representative of their usual levels of PA. Moreover, we assessed PA via the self-report IPAQ questionnaire, which requires participants to recall past activity, and is therefore, a subjective means of estimating individual PA levels that may be influenced by recall bias and social desirability bias. However, this design provides with the real-time information regarding the PA status of the BC population, required for designing pragmatic interventions that can be delivered within the NHS. A Limitation of our study is that we do not have data on age of individual patients attending for the NHSBSP. The ethics approval allowed us to collect only fully annonymised data from these patients. This cohort was aged between 50 and 69 years.

A further limitation of our study is that we cannot be sure that these findings are generalisable to women attending breast screening and women who are receiving or have completed PA treatment because our sample included only women from the Black Country area of the West Midlands in the UK. Our samples of chemotherapy and post-treatment BC patients were also relatively small, which further limits our ability to generalise. The NHSBSP sample was chosen as a “pre-diagnosis” comparison group based on their status as an “at risk of BC” group. However, clearly not all of the patients sampled will develop BC, and therefore, this group may not be representative of BC patients pre-diagnosis. However, this group is representative of women, who have the highest incidence of BC in the UK. Finally, BMI in the group of screening attendees was self-reported. This may have influenced the data of the present study; however, the effects of PA on BMI and/or the effects of the different stages of BC were not amongst the main aims of the present study.

In summary, we found that BC patients receiving chemotherapy had lower levels of PA compared to women attending breast screening and BC patients within one-year post-treatment, while post-treatment BC survivors had lower levels of PA compared to the breast screening group. The relatively low PA levels of the post-treatment BC survivors means that a large proportion of this group were not sufficiently exposed to the potential benefits of PA on BC survival. The findings of this current study, suggests the need to establish robust PA interventions to enhance the PA behaviour of post-treatment BC survivors at a time when the negative effects of chemotherapy begin to resolve and patients are more receptive to interventions designed to enhance their PA levels.
